# Cleavage Patterns of 9600 Embryos: The Importance of Irregular Cleavage

**DOI:** 10.3390/jcm12175656

**Published:** 2023-08-30

**Authors:** Maya Shavit, Daniel Gonen, Yuval Atzmon, Nardin Aslih, Asaf Bilgory, Yasmin Shibli Abu-Raya, Moamina Sharqawi, Daniela Estrada Garcia, Mediea Michaeli, Diana Polotov, Einat Shalom-Paz

**Affiliations:** 1 IVF Unit, Department of Obstetrics and Gynecology, Hillel-Yaffe Medical Center, Hadera 3810000, Israelmo2mena-9@hotmail.com (M.S.); einatshalompaz@gmail.com (E.S.-P.); 2 Ruth and Bruce Rappaport School of Medicine, Technion—Israel Institute of Technology, Haifa 3109601, Israel

**Keywords:** irregular cleavage, direct cleavage, clinical pregnancy rate, live birth rate, time-lapse imaging, morphokinetics

## Abstract

This prospective, observational study investigated the incidence of irregular cleavage (IRC) among human embryos and its influence on IVF treatment outcomes. It included 1001 women who underwent 1976 assisted reproduction treatments during 2016–2021 in a single IVF clinic. Embryo morphokinetics were analyzed and evaluated for the association between IRC and women’s characteristics, treatment characteristics, and pregnancy outcomes. The incidence of IRC was 17.5% (1689/9632 embryos). Of these, 85% of the embryos had one IRC, 15% had multiple IRC and 35% of IRC events occurred during the embryo’s first cell cycle. IRC embryos were found to correlate with male factor (*p* = 0.01) and higher ICSI rate (*p* = 0.01). Age, BMI, parity, basal FSH level, stimulation protocol, and number of retrieved oocytes did not differ between groups. Embryos with early IRC or more than one IRC had lower blastulation rates (*p* = 0.01 for each). Fresh cycles with IRC embryos had a lower clinical pregnancy rate (*p* = 0.01) and embryos with early IRC had a lower live birth rate (*p* = 0.04) compared to embryos without IRC. Frozen transfer cycles of blastocyst embryos, with or without IRC, had comparable results. In conclusion, the number of abnormal cleavage events and their timing are important factors in the prognosis of the developing human embryo.

## 1. Introduction

Selecting embryos with the greatest implantation potential is critical for achieving higher live birth rates and reducing both unsuccessful transfers and multiple birth rates in IVF cycles. Current research also focuses on improving the detection of euploid embryos. The two most important factors for successful implantation of the embryo are optimal development of the endometrium and embryo quality. As single embryo transfer is currently the desired standard, the need for optimizing embryo selection techniques has increased.

The techniques currently used for embryo selection are subjective and inconsistent and may lead to inaccurate embryo selection. Currently, there are two accepted methods for embryo selection. The traditional method based on morphology is known to be subjective and is associated with high intra- and inter-personal disagreement among embryologists [1]. There is also a variety of scoring systems in which the evaluation is based on assessing embryo development from a static point of view at fixed intervals. As a result, this technique is imprecise [2]. Morphokinetic assessment and analysis of human embryo development preimplantation, using time-lapse monitoring allows embryos to be reviewed at any point. Implementing the use of artificial intelligence technology in this dynamic review of embryo development may further provide indicative and objective data on embryo quality and implantation potential [3,4,5,6,7].

The increasing use of time-lapse monitoring systems provides qualitative data regarding embryo cleavage patterns and their relation to embryonic viability and implantation rates. Irregular cleavage (IRC) is defined as abnormal cell cleavage in the form of direct cleavage of a mother cell to more than two daughter cells.

In the last decade, numerous studies have focused on the search for morphokinetic markers that might be relevant to embryo viability and competency, and which could become potential biomarkers for embryo evaluation and selection, but the clinical benefits of morphokinetics have not yet been established [8]. However, only a few studies about embryo cleavage patterns have been published [9,10,11,12,13], all of which were retrospective and with relatively small sample sizes. In general, embryos with an IRC pattern are associated with lower developmental potential [9,14,15,16,17]. Furthermore, results from studies published thus far are contradictory regarding euploidy, implantation, and live birth rates. For example, Zhan et al. [13] reported that IRC embryos have a lower euploid rate in comparison to non-IRC embryos, but a similar live birth rate (LBR), while Ozbek et al. [9] reported the opposite. In a cohort of IRC embryos, the euploidy rate was higher in comparison to non-IRC embryos and the LBR was lower.

The purpose of this study was to contribute information to the current literature regarding the incidence of embryos with IRC, correlations to patient and treatment characteristics, and to compare IVF treatment outcomes between embryos with regular or irregular cleavage patterns.

## 2. Materials and Methods

This prospective, observational, cohort study was conducted at the IVF Unit of Hillel-Yaffe Medical Center in Israel, from January 2016 to December 2021.

### 2.1. Patients

All patients whose embryos were assessed with time-lapse technology for morphological and morphokinetic developmental patterns during fresh embryo development were included in the study. Cycles in which embryos were transferred on day 2, or more than 3 embryos were transferred, were excluded.

Demographic characteristics of the women enrolled, including age, parity, BMI, smoking history, basal FSH level, and infertility cause were collected from electronic medical records. Ovarian stimulation protocol (long GNRH agonist or GNRH antagonist), ovulation trigger (GNRH agonist or hCG), number of retrieved oocytes, and ICSI rate were analyzed.

IRC is defined as an abnormal cell cleavage in the form of a direct cleavage of a mother cell to more than two daughter cells regardless of the cell size (for example, one cell divided into three cells in a single division cycle). Only cells containing visible nuclei were considered blastomeres; otherwise, they were annotated as fragments [9,14,15,16,17].

Reverse cleavage was not evaluated in this study. We distinguished between early IRC (occurred in the first 24 h from the presence of 2PN) and late IRC (up to the third cleavage). IRC was followed only up to the third cleavage because of the technical difficulty of following IRC in a larger embryo with more cells. We also distinguished between single and multiple events of IRC. The analysis was based on these IRC characteristics. All embryos were assessed for IRC before deciding whether to transfer, freeze, or discard them. This information was calculated as part of the embryo’s final score, regardless of the destination of each embryo.

Outcomes of fresh and frozen transfer cycles were analyzed according to embryo stage at transfer (day 3 or day 5) and number of embryos per transfer (1 to 3). The day of embryo transfer and number of embryos transferred were determined based on patient age, history of past IVF treatments, and quality of embryos, either at the cleavage stage or on day 5 (blastocysts). Embryos were selected for transfer based on their KIDScore™, according to morphological evaluation. The KIDScore™ model is embedded in the annotation software and offered as a tool for the deselection of embryos showing abnormal morphokinetic patterns. The model is based on cumulative known clinical outcomes from many IVF centers [18]. The protocol at our clinic is to transfer cleavage-stage embryos as the first choice and to freeze up to 2 good-quality cleavage-stage embryos. The remaining high-quality embryos are cultured to blastocyst and frozen if they are good quality. All good-quality embryos that were frozen were cryopreserved in an open system, and the same freezing medium was used for all embryos.

### 2.2. Outcome Measures

Outcomes included morphokinetic parameters of multinucleation, fragmentation, time to blastulation, uneven blastomere percentile, and blastulation rate. Multinucleation was assessed at the 4-cell stage and presented as a score (from 0 to 4) of the number of blastomeres with multinucleation. Fragmentation was assessed at 2-cell and 4-cell stages and presented as a percentile.

Clinical outcomes included rate of high-quality embryos remaining for use (frozen or transferred), clinical pregnancy rate (CPR), which was defined as ultrasound confirmation of an intrauterine gestational sac with a heartbeat, LBR, miscarriage rate (spontaneous termination before 24 weeks of gestation), preterm labor rate (live deliveries occurring earlier than 37 weeks of pregnancy), and complication rates during gestation, including gestational hypertension, pre-eclampsia, abruption, intrauterine growth restriction, or intrauterine fetal demise.

### 2.3. Ethics

The Institutional Review Board (Helsinki Committee) of Hillel-Yaffe Medical Center approved the study protocol (0026-20-HYMC) on 27 April 2020. As the protocol involved only the observational review of patient charts, the need for individual patient informed consent was waived. All methods in the present study were performed strictly in accordance with the Declaration of Helsinki for Medical Research.

### 2.4. Data Analysis

Statistical analysis was performed using SPSS-22.0 for Windows (IBM Corp., Armonk, NY, USA). Categorical variables were analyzed using chi-squared or Fisher’s exact test. Continuous variables were analyzed using a *t*-test. For results that were significant or showed a statistical trend in univariate analysis, multivariable analysis was performed with a multiple logistic regression model to assess the potential impact of these parameters and to further investigate and determine the morphokinetic parameters and patient characteristics predictive of a successful or unsuccessful pregnancy outcome, CPR, and LBR. A *p*-value < 0.05 was considered statistically significant. All statistical tests were two-tailed. We also conducted a logistic regression for nominal parameters.

## 3. Results

Clinical outcomes were evaluated for 1001 patients undergoing 1976 IVF cycles, with 23,605 retrieved oocytes, and 9632 developed embryos. The reasons for infertility were 38.9% male factor, 27% unexplained infertility, 11% tubal factor, 8% anovulation, 3% endometriosis, and 10.5% other causes. The women underwent a mean of 1.97 cycles. The mean number of oocytes per patient per treatment was 11.94 ± 7.38.

The incidence of IRC embryos was 17.5% (1689/9632). Of these, 35% had early IRC (occurring during the embryo’s first cell cycle) and 65% had late IRC (occurring after the embryo’s first cell cycle ended). In addition, 7943 (82.5%) embryos had no IRC, 1447 (15%) embryos had one IRC, and 242 (2.5%) embryos had two or more IRC.

### 3.1. Patient Characteristics

Age, BMI, parity, and basal FSH level were not correlated with the number of IRC events or with their timing. IRC embryos were found to correlate with male factor infertility (35.7% vs. 31.2%, *p* = 0.01; Table 1 and Table 2).

### 3.2. Treatment Characteristics

Neither the number of IRC events nor their timing was influenced by the stimulation protocol, trigger medication, or number of retrieved oocytes. The ICSI rate was higher in IRC embryos in comparison to non-IRC embryos (89% vs. 87%, *p* = 0.01; Table 1 and Table 2).

### 3.3. Other Morphokinetic Parameters

Both a higher number of IRC events during the embryo’s development course and earlier time to the first IRC event were found to correlate with higher rates of multi-nucleation and uneven blastomere size. An early IRC event also correlated with a higher fragmentation rate (14.9%, 18%, and 12% for none, early and late IRC events, respectively, *p* < 0.01; Table 3 and Table 4).

### 3.4. IVF Outcomes

#### 3.4.1. Blastulation

Blastulation rate was lower in the group with two or more IRC (11.4%) compared with no (19.9%) or one (20.8%) IRC only (*p* = 0.01). The Blastulation rate was also lower when comparing early IRC (15.6%) to no (19.9%) or late IRC (21.7%) (*p* = 0.01). Also of importance is that the time to blastulation was longer for IRC embryos in comparison to those without (Table 3 and Table 4).

#### 3.4.2. Rate of Usable Embryos

The usable embryo rate was defined as embryos chosen for either transfer or freeze. The rate was significantly lower for embryos with IRC (53.9%) and even more so for embryos with two or more IRC (14% and 9.5%, respectively, *p* < 0.01). The rate was also lower for embryos with early vs. late IRC (9.0% vs. 15.9%, *p* < 0.01; Table 3 and Table 4).

#### 3.4.3. Clinical Pregnancy, Live Birth, and Miscarriage Rates in Fresh Cycles

An IRC embryo was transferred in 133 fresh cycles. All fresh transfer cycles (cleavage stage and blastocyst) were combined for outcome analysis due to the small sample size. Among these, 23 were single embryo transfers (SET), and 2 or 3 embryos were transferred in the remaining 110 cycles. In most cycles with multiple embryos, at least one did not have an IRC event. The CPR per transfer decreased as the number of IRC events increased (30.8% for none, 23.4% for one, and 5.0% for two or more IRC events; *p* = 0.01). Embryo transfer (ET) with an early IRC embryo had both lower CPR (9.4%, 23.7%, 30.8%; *p* = 0.01) and lower LBR (3.1%, 15.8%. 20.5%; *p* = 0.04) compared with late and no IRC. Miscarriage rates did not differ significantly between the study groups (Table 3 and Table 4).

#### 3.4.4. Preterm Labor and Placental Complications in Fresh Cycles

Preterm labor was significantly more prevalent in the IRC groups (23% in the single IRC event group and 25% in the late IRC groups vs. 10% in the no IRC group, *p* = 0.04 and 0.02, respectively). There were no preterm deliveries in the group of early or more than one IRC event group. No significant difference was found in the rate of placental complications between the study groups (Table 3 and Table 4).

#### 3.4.5. IRC Frozen Embryo Transfer Outcomes

A total of 95 embryos with IRC were frozen, all at the blastocyst stage. Of these embryos, 51 were transferred in a frozen cycle, of which 32 were SET and 19 were dual embryo transfers (DET). In the DET cycles, the transferred embryo included one embryo with an IRC event and the other had normal cleavage. The CPR of these cycles was 25% (8/32) in the SET cycles and 36% (7/19) in the DET cycles (*p* = 0.369). In comparison, CPR for SET of a blastocyst without IRC in a frozen cycle was 37% (*p* = 0.17) and LBR was 22% (*p* = 0.37). We cannot compare DET for blastocysts without IRC as the protocol is to transfer only one blastocyst when there is no IRC. The LBR was 15.6% (5/32) in the SET cycles and 10.5% (2/19) in the DET cycles (*p* = 0.61; Table 5).

### 3.5. Logistic Regression

Logistic regression analysis to determine the variables that contributed to the LBR, found only younger age and higher parity to be significant parameters for achieving both clinical pregnancy and a live birth. BMI, duration of infertility, etiology of infertility, number of oocytes retrieved, time to cleavage stage embryo, degree of embryo fragmentation, embryo degree of multi-nucleation, and number of IRC events and their timing were not related to achieving clinical pregnancy or live delivery (Table 6).

## 4. Discussion

The present study evaluated the impact of IRC on IVF treatment and pregnancy outcomes. The information obtained contributes to the existing yet meager literature regarding the reproductive potential of embryos with no, single, or multiple IRC events and their timing [9,10,11,12,13,15,16,17]. We demonstrated that male factor and ICSI correlate with IRC, and that a single early IRC event or multiple occasions of IRC have deleterious effects on embryo development and on IVF outcomes in terms of blastulation rate. However, the blastulation rate was not affected by a single late IRC event. Unlike the blastulation rate, the CPR was found to be significantly lower in cases with a single late IRC and even more so in cases with an early IRC or multiple instances of IRC. We found that frozen blastocysts with IRC could lead to pregnancies, although the CPR was lower in comparison to FET with normal cleavage embryos. To the best of our knowledge, this is the first study to follow these pregnancies until delivery and investigate preterm labor and placental complications in pregnancies resulting from an ET of an IRC.

Analysis of IRC events in relation to the cause of infertility revealed a connection between sperm defects and IRC. ICSI was used more frequently with the single and late IRC embryo groups. One hypothesis underlying IRC formation is that it is caused by multipolar spindles through the introduction of either incomplete, defective, or supernumerary centrioles of defective sperm [19]. Another hypothesis that we suggest is that the ICSI procedure itself might damage the miotic spindle of the ovum, which in turn will manifest as IRC. However, this finding contradicts a previous study [13] that found a slightly higher single IRC rate in the group of IVF-fertilized embryos compared to the ICSI-fertilized group (11.5% vs. 9.1%). Interestingly, this difference was not found in the group of multiple IRC embryos where there was no significant correlation between fertilization method and IRC. A possible explanation for the contradictory findings might be that the cohort size was not large enough to truly represent the nature of the phenomenon or was due to natural variability in the causes of IRC in different populations.

The same study [13] also reported that sperm origin influences embryo cleavage patterns. Testicular/epididymal sperm correlated with a higher IRC rate than ejaculated sperm.

The impact of paternal age is debated in the literature. Ozbek et al. [9] found a correlation between advanced paternal age and IRC, while Zhan et al. [13] did not. It will be interesting to re-examine our findings of ICSI fertilization as a contributing factor to higher IRC rates in a larger study. There are still many questions to be answered regarding the link between the male factor and IRC embryos. For example, further investigation of whether certain semen parameters, such as DNA fragmentation or teratospermia are predictive of IRC.

The impact of ovarian response on IRC has been discussed previously. A study by Alexopoulou et al. [20] evaluated the incidence of IRC in patients with low vs. normal response. They reported no statistically significant difference in morphokinetic parameters other than time t3 (dif = 0.884; *p*-value < 0.046), and no significant differences were observed in irregular cleavage patterns, multinucleation at the two-cell stage, fragmentation, or non-tetrahedral shape rate at the four-cell stage. These results are similar to those of our study. We found no connection between IRC and ovarian response in terms of the number of retrieved oocytes or patients’ basal FSH levels.

In regard to blastulation rate in IRC embryos, similar to previous studies [12,13], we found lower blastulation rates for embryos with early or multiple IRC events. However, a lower blastulation rate was not demonstrated for embryos with single and/or late IRC. This is most likely due to a less deleterious effect of the IRC at later developmental stages of the embryo.

As previously mentioned, early compared to late IRC, has a detrimental effect on embryonic development. This is manifested by lower blastulation rates, fewer usable embryos, and lower clinical pregnancy and live birth rates.

The molecular mechanisms that underlie IRC are not entirely clear, but it is widely accepted that mitotic errors play a vital role in irregular division. When IRC occurs late, there is at least one blastomere that cleaves normally and contributes to the chromosomal balance of the embryo, thereby bolstering its viability and eventually leading to higher implantation and live birth rates. Lagalla et al. [10] explored the possibility of a potential “aneuploidy rescue” mechanism, as they observed that IRC embryos that excreted some cells during the compaction process developed into euploid blastocysts. We believe that further genetic analysis could reveal new information about the differences in the self-correction mechanisms between early and late IRC and about the molecular underpinnings of both abnormal cleavage patterns.

A few recent studies included pregestational genetic analysis of the IRC embryos. These studies primarily included patients with poor prognoses: advanced maternal age, severe male factor, recurrent unexplained pregnancy loss, and repeated implantation failure. Most of the embryo biopsies reported were performed at the blastocyst stage and only some of the studies mentioned the timing of the IRC event during embryonic development. Lagalla et al. [10] and Zhan et al. [13] reported that the euploidy rate gradually increased when the IRC event occurred later. Surprisingly, IRC embryos were found to have euploid rates comparable to those of embryos without IRC. Ozbek et al. [9] reported that IRC embryos even had a higher rate of euploidy in comparison to non-IRC embryos, although without mentioning the timing of the IRC. Unfortunately, genetic analysis of the embryos was not available in the current study.

Interestingly, we found a higher rate of preterm labor in the late IRC group. To the best of our knowledge, this is the first study to investigate obstetric complications, including preterm birth in pregnancies developed from IRC embryos. There were no preterm deliveries in the early or more than one IRC event group, but this is most likely probabilistic given the small size of the groups. The finding of a higher preterm birth rate in correlation to IRC embryos should be confirmed by further studies.

A higher number of IRC events during embryonic development represents more mitotic errors and fewer blastomeres that cleave normally. Only one study [13] mentioned this issue. In agreement with Zhan et al. [13], we also found lower blastulation rates and lower CPR in the group with multiple IRC events. Moreover, we found fewer usable embryos and a higher preterm birth rate in this group. Zhan et al. [13] performed genetic analyses and did not find significant differences between one vs. multiple IRC events.

We also compared the outcomes of frozen transfers of embryos with and without IRC. In general, embryo cryopreservation enabled the conservation of surplus embryos for further use, thus increasing the cumulative LBR after an IVF cycle. Previous studies have shown higher pregnancy rates and better perinatal outcomes with frozen embryo transfer than with fresh embryo transfer. It has been hypothesized that frozen embryo transfer may provide a more favorable intrauterine environment for embryo implantation and placentation by avoiding the supraphysiologic condition that occurs after ovarian stimulation [21]. In our unit, we use the open vitrification system for vitrification of all embryos [22]. Given recent studies, including one from our unit, ovulatory cycles are preferred over artificial cycles in terms of pregnancy rate, delivery rate, and lower complication rate [23]. Importantly, long-term outcomes of frozen embryo transfer cycles were also evaluated and found to be comparable with fresh cycles in terms of congenital malformation rate, neurodevelopmental disorders, growth, and chronic diseases [24]. Our unit policy regarding surplus IRC embryos is to follow up on their development until the blastocyst stage. We assume that if the IRC embryo has already developed into a good-quality blastocyst, its morphology at the cleavage stage does not impact the ongoing pregnancy rate greatly, as based on a previously published work [25]. Indeed, as we assumed, we found that IRC embryos achieving the blastocyst stage had comparable clinical pregnancy and live birth rates to normal cleavage embryos. These results are similar to those in the studies mentioned above [9,10].

The strengths of the present study include a relatively large sample size of human embryos incubated in EmbryoScope^®^, in a single IVF center with a limited number of embryologists; all with high levels of expertise, and homogeneous guidelines regarding embryo estimation and management. All IRC annotations were confirmed by the embryologists. This was a prospective observational study; hence, the data are very accurate. Only the data analysis was performed retrospectively.

This study had a few limitations. First, although the number of embryos produced and analyzed was quite high, the number of IRC embryos transferred was not as high due to our departmental policy, which prefers the transfer of normal cleavage embryos. Another limitation was that the embryos transferred did not undergo genetic analysis. And yet another real drawback was the number of embryos transferred per cycle. In many cycles with an IRC embryo, a parallel, normally cleaved embryo was transferred. This makes it more difficult to interpret the CPR and LBR.

In conclusion, given the results of this and previous studies, embryos with an IRC pattern should be given lower priority for transfer, mainly in the cleavage stage. Since the LBR after transfer of an IRC embryo is significantly lower compared with normal cleavage embryos, we recommend that blastocysts with irregular cleavage patterns should be considered a second-choice candidate for ET if they are morphologically suitable.

## Figures and Tables

**Table 1:** Patient and treatment characteristics by number of irregular cleavage events during embryo development.

Characteristic	No IRC (n = 7943)	1 IRC (n = 1447)	2 + IRC (n = 242)	*p* Value
Maternal age (years ± SD)	34.2 ± 6.5	33.9 ± 6.3	34.1 ± 6.2	0.336
Parity (mean ± SD)	0.4 ± 0.7	0.4 ± 0.6	0.4 ± 0.6	0.207
BMI (kg/m^2^ ± SD)	26.3 ± 6.2	26.5 ± 6.0	25.5 ± 5.9	0.214
Basal FSH level (mean ± SD)	7.7 ± 4.0	7.6 ± 3.6	7.9 ± 3.9	0.673
Infertility cause (n (%))				
Anovulation	824 (10.4%)	127 (8.8%)	21 (8.7%)	0.136
Endometriosis	213 (2.7%)	38 (2.6%)	2 (0.8%)	0.206
Male factor	2480 (31.2%)	520 (35.9%)	84 (34.7%)	0.001
Tubal factor	980 (12.3%)	188 (13%)	25 (10.3%)	0.48
Unexplained	1552 (19.5%)	278 (19.2%)	59 (24.4%)	0.16
Ovarian stimulation protocol, n (%)				
Long GNRH agonist	786 (9.8%)	168 (11.6%)	29 (12%)	
GNRH antagonist	5922 (74.6%)	1043 (72.1%)	178 (73.6%)	0.199
Other	1235 (15.5%)	236 (16.3%)	35 (14.4%)	
GNRH agonist ovulation trigger, n (%)	802 (10.1%)	162 (11.2%)	22 (9.1%)	0.375
Number retrieved oocytes (mean ± SD)	14.5 ± 9.3	14.9 ± 8.7	14.3 ± 8.2	0.315
ICSI (n (%)	6910 (87%)	1301 (90%)	208 (86%)	0.007

IRC, irregular cleavage; SD, standard deviation; BMI, Body mass index; ICSI, intracytoplasmic sperm injection.

**Table 2:** Patient and treatment characteristics by timing of first irregular cleavage event during embryo development.

Characteristic	No IRC (n = 7943)	Early IRC (n = 588)	Late IRC (n = 1,101)	*p* Value
Maternal age (years ± SD)	34.2 ± 6.5	33.7 ± 6.3	34.1 ± 6.3	0.169
Parity (mean ± SD)	0.4 ± 0.7	0.4 ± 0.7	0.4 ± 0.6	0.175
BMI (kg/m^2^ ± SD)	26.3 ± 6.2	25.8 ± 6.0	26.3 ± 6.0	0.178
Basal FSH level (mean ± SD)	7.7 ± 4.0	7.8 ± 4.0	7.5 ± 3.1	0.534
Infertility cause (n (%))				
Anovulation	824 (10.4%)	58 (9.9%)	90 (8.2%)	0.074
Endometriosis	213 (2.7%)	18 (3.1%)	22 (2.0%)	0.328
Male factor	2480 (31.2%)	208 (35.4%)	396 (36%)	0.001
Tubal factor	980 (12.3%)	72 (12.2%)	141 (12.8%)	0.901
Unexplained	1552 (19.5%)	114 (19.4%)	223 (20.3%)	0.846
Ovarian stimulation protocol, n (%)				
Long GNRH agonist	786 (9.8%)	70 (11.9%)	127 (11.5%)	
GNRH antagonist	5922 (74.6%)	421 (71.6%)	800 (72.7%)	0.224
Other	1235 (15.5%)	97 (16.4%)	174 (15.8%)	
GNRH agonist ovulation trigger, n (%)	(10.1%) 802	(13.1%) 77	(9.7%) 107	0.057
Retrieved oocytes (mean ± SD)	14.5 ± 9.3	15.0 ± 9.0	14.7 ± 8.5	0.383
ICSI, n, (%)	6910 (87%)	518 (88%)	991 (90%)	0.011

IRC, irregular cleavage; SD, standard deviation; BMI, Body mass index; ICSI, Intracytoplasmic sperm injection.

**Table 3:** IVF outcomes by number of IRC events during embryo development.

Characteristic	No IRC (n = 7943)	1 IRC (n = 1.44)	2 + IRC (n = 242)	*p* Value
Multi-nucleation (mean ± SD)	0.98 ± 0.86	1.3 ± 0.9	1.7 ± 0.8	<0.001
Fragmentation (%)	14.90%	14.30%	13.20%	0.128
Uneven blastomere, n (%)	2224 (28%)	651 (45%)	118 (49%)	<0.001
Blastocyst formation, n (%)	1581 (19.9%)	301 (20.8%)	26 (11.4%)	0.004
Time to blastulation (mean ± SD)	108.5 ± 10.1	112.4 ± 9.5	111.1 ± 8.9	<0.001
Usable embryos, n (%)	4280 (53.9%)	202 (14.0%)	23 (9.5%)	<0.001
Fresh cycle outcomes				
Fresh cycles with ET, n (%)	1808 (22.7%)	113 (7.8%)	20 (8.2%)	<0.001
Clinical pregnancy, n (%)	562/1808 (30.8%)	26/113 (23.4%)	1 (5%)	0.009
Live birth, n (%)	356/1808 (20.5%)	16/113 (14.1%)	1 (5%)	0.095
Miscarriage, n (%)	127/562 (22%)	6/26 (23%)	0/1 (0%)	0.999
Preterm birth, n (%)	57/562 (10%)	6/26 (23%)	0/1 (0%)	0.037
Placental complications, n (%)	33/562 (6%)	0/26 (0%)	0/1 (0%)	0.091

IRC, irregular cleavage; SD, standard deviation; ET, embryo transfer.

**Table 4:** IVF outcomes according to timing of first IRC event during embryo development.

Characteristic	No IRC (n = 7943)	Early IRC (n = 588)	Late IRC (n = 1101)	*p* Value
Multi-nucleation (mean ± SD)	0.98 ± 0.86	1.61 ± 1.0	1.3 ± 0.8	<0.001
Fragmentation (%)	14.9%	18%	12%	<0.001
Uneven blastomere, n (%)	2224 (28%)	411 (70%)	385 (35%)	<0.001
Blastocyst formation, n (%)	1581 (19.9%)	92 (15.6%)	239 (21.7%)	0.011
Time to blastulation, minutes (mean ± SD)	108.5 ± 10.1	112.1 ± 10.6	112.4 ± 9.0	<0.001
Usable embryos, n (%)	4280 (53.9%)	53 (9.0%)	175 (15.9%)	<0.001
Fresh cycle outcomes				
Fresh cycles with ET, n (%)	1808 (22.7%)	32 (5.4%)	101 (9.2%)	<0.001
Clinical pregnancy, n (%)	(30.8%) 562/1808	3/32 (9.4%)	24 (23.7%)	0.010
Live birth, n (%)	(20.5%) 56/1808	1/32 (3.1%)	16 (15.8%)	0.042
Miscarriage, n (%)	(22%) 127/562	2/3 (66%)	3/24 (12.5%)	0.093
Preterm birth, n (%)	57/562 (10%)	0/3 (0%)	6/24 (25%)	0.021
Placental complication, n (%)	33/562 (6%)	0/3 (0%)	0/24 (0%)	0.105

IRC, irregular cleavage; SD, standard deviation; ET, embryo transfer.

**Table 5:** Irregular cleavage (IRC) frozen embryo transfer outcomes.

Characteristic	No IRC (n = 261)	IRC (n = 95)	*p* Value
Single embryo transfer			
Clinical pregnancy rate, n (%)	95/261 (37%)	8/32 (25%)	0.175
Live birth rate, n (%)	59/261 (22%)	5/32 (15.6%)	0.367
Dual embryo transfer			
Clinical pregnancy rate, n (%)	Not applicable	7/19 (36%)	N/A
Live birth rate, n (%)	Not applicable	2/19 (10.5%)	N/A

IRC, irregular cleavage; N/A, Not applicable.

**Table 6:** Multivariable logistic regression analysis for clinical pregnancy rate.

Variable	Odds Ratio	95% CI for Odds Ratio		*p* Value
		Lower	Upper	
Age	0.928	0.907	0.949	<0.001
Parity	1.274	1.053	1.542	0.013

CI, confidence interval.

## Data Availability

All datasets used and analyzed in this study are available from the corresponding author on request.
